# The experience of caring for patients at the end-of-life stage in non-palliative care settings: a qualitative study

**DOI:** 10.1186/s12904-018-0372-7

**Published:** 2018-10-17

**Authors:** Xiao Bin Lai, Frances Kam Yuet Wong, Shirley Siu Yin Ching

**Affiliations:** 1 School of Nursing, Fudan University, Shanghai, China; 2 School of Nursing, The Hong Kong Polytechnic University, Hung Hom, Hong Kong

**Keywords:** End-of-life care, Health care delivery, Qualitative research, Life-threatening diseases

## Abstract

**Background:**

More patients are dying in non-palliative care settings than in palliative care settings. How health care providers care for adult patients at the end-of-life stage in non-palliative care settings has not been adequately explored. The aim of this study was to explore the experiences of health care providers in caring for patients at the end-of-life stage in non-palliative care settings.

**Methods:**

This is a qualitative study. Twenty-six health care providers from eight health care institutions which are based in Shanghai were interviewed individually between August 2016 and February 2017. Three levels of health care, i.e., acute care, sub-acute care, or primary care, was provided in the health care institutions. The interviews were analyzed using qualitative content analysis.

**Results:**

Three themes emerged from the interviews: (i) Definition of the end-of-life stage: This is mainly defined based on a change in treatment. (ii) Health care at the end-of-life stage: Most patients spent their last weeks in tertiary/secondary hospitals, transferring from one location to another and receiving disease- and symptom-focused treatment. Family-dominated decision making was common when discussing treatment options. Nurses instinctively provided extra care attention to patients, but nursing care is still task-oriented. (iii) Challenges, difficulties, and the future. From the interviews, it was found that pressure from families was the main challenge faced by health care providers. Three urgent tasks before the end-of-life care can become widely available in the future were identified from the interviews, including educating the public on death, extending government support, and creating better health care environment.

**Conclusion:**

The end-of-life care system of the future should involve health care institutions at all levels, with established mechanisms of collaboration between institutions. Care should be delivered to patients with various life-threatening diseases in both palliative and non-palliative care settings. But first, it is necessary to address the obstacles to the development of end-of-life care, which involve health care providers, patients and their families, and the health care system as a whole.

**Electronic supplementary material:**

The online version of this article (10.1186/s12904-018-0372-7) contains supplementary material, which is available to authorized users.

## Background

End-of-life care is that part of palliative care that focuses on patients who have life-threatening diseases and a life expectancy of 6 to 12 months [1, 2]. It helps patients to die in peace, comfort, and dignity [1]. Globally, the estimated number of people in need of end-of-life care is 20.4 million [1]. By 2040, an estimated 75% to 88% of patients at the end-of-life stage could benefit from palliative care [3]. All countries should take heed of the increasing need for end-of-life care [4].

Patients with life-threatening diseases at the end-of-life stage suffer multiple physical discomforts [5, 6], as well as a range of psychosocial and spiritual concerns [7–12]. With the progression of their diseases, the utilization of health services by patients increases at the end-of-life stage [13–19]. As a result, worldwide, a significantly large number of patients with chronic diseases (20–78%) die in acute care hospitals [20–22]. Yet acute care hospitals may not have specialized palliative care or hospice care wards. With the huge number of patients at the end-of-life stage, more patients are dying in non- palliative care settings than in specialized hospice care wards in hospitals or hospice facilities [23, 24]. A nation-wide survey conducted in the United Kingdom (UK) reported that only 6% of those who passed away died in hospice facilities [25].

The situation in China is consistent with the international trend. The health care system in China has been undergoing reform for years. During the previous reform period (from the early 1980s to the 2000s), China dismantled the country’s effective health care and public health system, which caused much turmoil years later [26]. One of the negative outcomes of this move has been the decentralization of the public health system, which has led to great disparities between rural and urban health care service [26]. Patients tend to bypass local health care institutions to seek care in the larger acute care hospitals in urban areas, even for relatively minor problems. As of the year 2017, there were 989,000 health care institutions in China, of which 30,000 were hospitals and 934,000 were primary health care institutions [27]. That same year, there were a total of 660 million health care visits in China, of which, 280 million were to hospitals, 360 million to primary health care institutions [28]. The hospitals have been overused for years.

Patients with chronic diseases usually go to large acute care hospitals to seek help. When their condition deteriorates, patients will stay in a hospital until they passed away. Previous studies found that more than half of patients at the end-of-life stage in Beijing, Shanghai, and Guangdong died in an acute care hospital [29–31]. Hospice care in China has developed rapidly since the announcement of several national policies on end-of-life care in 2015 [32–34]. Yet the care provided to dying patients is far from satisfaction. According to the 2015 Quality of Death Index report, mainland China ranked 71st out 80 countries and areas in its quality of death [35]. Before the wide introduction of the concept of hospice care, only 1% of patients at the end-of-life stage in China received hospice care [36]. The rate of utilizations of hospice care services is currently still low. In Shanghai, for example, a community-based hospice service was established with a great deal of support from the government, and the utilization of hospice beds was 43.8% [37]. However, only 11.8% of patients at the end-of-life stage died in hospice wards in the community [37].

In the face of the increasing need for palliative care, the World Health Organization and the Worldwide Palliative Care Alliance proposed that palliative care be delivered at all levels of care: (i) by all health care providers through a “palliative care approach”; (ii) by primary health care providers and those treating patients with life-threatening diseases, who would provide “general palliative care” and; (iii) by specialized teams treating patients with complex problems, who would provide “specialist palliative care” [1]. Palliative care should be provided wherever a person’s care takes place [1]. Hence, as part of palliative care, it is necessary to develop and improve end-of-life care in non-palliative care settings.

Patients at the end of their life and living in non-palliative care settings may not be receiving care appropriate for their stage of illness [38, 39]. A review article found that the management of symptoms for dying patients was not adequate in many hospitals [39]. In the UK, nearly one-third (31%) of the bereaved families were not satisfied with the care that was provided in the hospital where a family member was a patient during the last 3 months of his/her life [25]. By comparison, the proportion of bereaved families who were dissatisfied with the care provided in hospice facilities, in their own home, and in care homes was 21%, 21%, and 18%, respectively [25]. Studies conducted in the United States (US), the UK, and the Netherlands found that the quality of dying and death in hospitals is not satisfactory [25, 40, 41]. Thus, how to enhance the end-of-life care in various clinical settings is a challenge faced by health care professionals.

Identifying gaps in service is a prerequisite to developing end-of-life care in non-palliative care settings. It is essential to know how patients at the end-of-life stage are cared for in hospitals from the perspectives of both health care providers and health care recipients. In the study by McIlvennan et al., the families felt that the hospice care provided to patients with a left ventricular assistance device was fragmented [42]. Peacock et al. reported that the family caregivers of dementia patients thought that physicians specializing in treating dementia patients did not seem like the idea of end-of-life care [43]. A recent review article suggested that a holistic approach be taken to providing end-of-life care to patients [44].

Patients at the end-of-life stage and their family caregivers have unmet needs in multiple domains. Mathews and Johnston identified the unmet needs of people with chronic obstructive pulmonary disease (COPD) and their caregivers as being in the physical, psychosocial and spiritual domains [45]. Of particular importance are appropriate and timely conversations to identify the personal preferences of patients at the end of their life and to provide relevant information on COPD [45]. The need for better communication with health care providers was also reported in another study [46]. Spending time and discussing important issues with patients have also been identified as family’s needs [8, 47]. Clark et al. [8] found that family members with a relative who had died in an acute medical ward wished that they had been able to spend more time with the patient and address issues before he/she passed away. Mossin and Landmark’s study [47] also found that families wanted to spend time with patients. The necessity of communicating with patients about death and important issues is echoed in another study on family members [48]. Family members also need more information and good support from health care providers, and good symptom management for patients [8].

Compared with the research that has been conducted on family caregivers, how health care providers care for adult patients at the end-of-life stage, especially in non-palliative care settings, has not been adequately explored. Garner et al. [49] found that, from the perspective of nurse executives, inadequate communication, lack of education, and hospital system constraints were three major barriers to providing end-of-life care in hospital settings. Another study found that, nurses considered it a challenge to provide end-of-life care in the complex and difficult environment of the medical units [50]. In their study, Gardiner et al. discovered that attitudinal differences to the care, a focus on curative treatments, and a lack of resources were the main barriers to providing palliative care for older people in acute care hospitals [51]. These studies explored the experiences of nurses in caring for patients at the end-of-life stage. Few studies have presented a comprehensive picture of how patients at the end-of-life stage are cared for in hospitals from the perspective of both physicians and nurses. Therefore, in this study the following research question was put forward: What have been the experiences of health care providers in caring for patients at the end-of-life stage in non-palliative care settings?

## Methods

### Aims

The aim of the study was to explore the experiences of health care providers in caring for patients at the end-of-life stage in non-palliative care settings.

### Design

The experiences being examined in this study came from the health care providers’ engagement in the health care system and their social interactions with patients and their families. The answer to the research question emerged through the dialogues that took place between the researcher and the participants. Hence, we aligned ourselves with an interpretivist perspective. Data were collected through interviews and analyzed using qualitative content analysis.

### Participants

The criteria for the inclusion of participants were: (a) being a physician or a nurse working in a health care institution in Shanghai, where in a tertiary hospital (i.e., an acute care hospital), a secondary hospital (i.e., a sub-acute care hospital), or community health care center; (b) working in an internal medicine ward, intensive care unit (ICU), or acute and emergency department (AED); and (c) having cared for adult patients at the end-of-life stage during the past two years. The patients were those with cancer or a non-cancer chronic disease. Physicians or nurses who worked in palliative care units were excluded.

Purposive sampling was adopted to ensure that participants working in various wards could be interviewed. An advertisement was sent out via WeChat, the biggest social media application in China [52], with 963 million users in the country [53]. The advertisement of this project was sent to people on WeChat who were acquaintances of the first author (X.B.L.). It was estimated that more than 300 people saw the advertisement. Eligible health care providers were introduced to the first author (X.B.L.) by friends and colleagues if they expressed an interest in the research topic. X.B.L. then sent the information sheet to the eligible health care providers at least one week before the interviews. Any concerns were answered immediately. The interviews were confirmed after the health care providers agreed to participate. The consent form was signed on the day of the interview after the participant was given a face-to-face introduction to the study.

### Data collection

The data were collected between August 2016 and February 2017. Semi-structured individual interviews were conducted by X.B.L. using an interview guide (see Table 1) in quiet rooms in the wards. Each participant was interviewed once. A minor revision was made to the interview guide after it was pilot tested in two interviews that were not included in the data analysis. The interviewer is an academic staff member with a PhD, who has a great deal of experience in conducting interviews. All of the interviews were audio taped and averaged 41 min in length. Participant recruitment was stopped when data saturation was achieved [54]. Data saturation was determined using the criterion of “informational redundancy” proposed by Grady and Sandelowski [55, 56]. When the researcher began to hear the same comments again and again in the interviews, data saturation was reached.

**Table 1:** Interview Guide

Interview questions	
1. Please enlighten me on the trajectory of disease(s) in your specialty. From your point of view, how would you define the end-of-life stage of the disease?	
2. What kinds of treatment/care have you provided to patients at this stage?	
3. What challenges/difficulties have you experienced when caring for such patients?	
4. From your point of view, how should these patients be cared for?	

### Ethical considerations

Ethical approval was obtained from the ethics committee of the university in which the first author works before the study began (Reference number: IRB#2017-10-1). The informants were assured that their participation was voluntary and confidential. Each participant was identified by a research number. All of the participants signed the consent form before the interviews were conducted.

### Data analyses

The interviews were transcribed verbatim in Mandarin. A transcript was sent to one participant to check against with the audio record for accuracy. The transcripts were then imported into NVivo software [57] and analyzed using qualitative content analysis [58]. The analytic steps included: (a) thoroughly reading the transcripts; (b) dividing the interviews into *content areas* according to the interview questions; (c) extracting and condensing *meaning units*; (d) abstracting and labeling a *code*; (e) sorting the codes into *categories*; and (f) creating *sub-themes/themes* [58]. The data were mainly coded by the first author and reviewed by the other two authors. The analytic results were discussed by all three authors.

### Rigor

The rigor of a study refers to its credibility, transferability, dependability, and conformability [59]. Health care providers in different specialties at several institutions were interviewed as a way of triangulating sources of data [60]. In each ward, one physician and one nurse were interviewed. Triangulation through multiple analyses was also performed [60]. The three researchers, all academic staff members, reviewed the data set independently, held group discussions on the findings, addressed the differences in their views, and finally arrived at a consensus. One participant reviewed the findings as another approach to analytical triangulation [60]. Verbatim quotations were provided to boost the transferability and credibility of the study [59]. A personal journal was kept to record the researcher’s thoughts, including the frustrations, challenges, and highlights that were encountered while conducting the study [61].

## Results

Twenty-six health care providers from eight institutions were approached. All agreed to participate. Their demographic characteristics are listed in Table 2. Three themes emerged from the interviews: (i) Definition of the end-of-life stage; (ii) Health care at the end-of-life stage; (iii) Challenges, difficulties, and the future. An illustration of the coding process has been included as Additional file 1.

**Table 2:** Demographics of the Participants

	Physician (*N* = 13)	Nurse (*N* = 13)
Age (Mean [*SD*])	39.00 (9.28)	37.15 (4.34)
Gender (n [%])		
Female	5 (38.5)	13 (100)
Education (n [%])		
Tertiary school	5 (38.5)	12 (92.3)
Master’s degree	5 (38.5)	1 (7.7)
Doctoral degree	3 (23.0)	0 (0)
Religion (n [%])		
Buddhist	3 (23.0)	1 (7.7)
None	10 (76.9)	12 (92.3)
Marital status (n [%])		
Married	12 (92.3)	12 (92.3)
Divorced	1 (7.7)	0 (0)
Single	0 (0)	1 (7.7)
Working since graduation (year) (Median [range])	10 (5–37)	17 (4–25)
Working in this specialty (year) (Median [range])	10 (0.2–19)	11 (1–16)
Level of health care institution (n [%])		
Tertiary hospital	7 (53.8)	6 (46.2)
Secondary hospital	4 (30.8)	5 (38.5)
Community health care center	2 (15.4)	2 (15.4)
Specialty (n [%])		
AED	4 (30.8)	3 (23.1)
ICU	1 (7.7)	1 (7.7)
Cardiology department	2 (15.4)	2 (15.4)
Pulmonary department	0 (0)	2 (15.4)
Gastroenterology department	1 (7.7)	0 (0)
Hepatology department	1 (7.7)	1 (7.7)
Nephrology department	2 (15.4)	2 (15.4)
General practice	2 (15.4)	2 (15.4)
Diagnoses of patients cared for by the participants (n [%])	Total (*n* = 26)	
Cancer	22 (84.6)	
Cardiovascular disease	22 (84.6)	
Diabetes	11 (42.3)	
Chronic obstructive pulmonary disease	12 (46.2)	
Renal failure	16 (61.5)	
Cirrhosis	11 (42.3)	
Dementia	5 (19.2)	
Parkinson’s disease	7 (16.9)	
Multiple sclerosis	2 (7.7)	

### Definition of the end-of-life stage

The participants defined the end-of-life stage of a person who has a chronic disease mainly in terms of a change in treatment.

“A patient could be described as being at the end-of-life stage if he/she needs intravenous drugs to maintain (his/her physical status).” (Physician 15, cardiology)

Some few criteria for the end-of-life stage of those with specific diseases were also mentioned, including increased hospital admissions (Nurse 8, pulmonary), being bed-bound all day (Nurse 10, cardiology), and having a Creatinine level over 400 (Physician 16, nephrology). They stated that the duration of the end-of-life stage varies from days to months, and is unpredictable.

### Health care at the end-of-life stage

#### Hospitalization: Drifting in the health care system

The need for hospitalization increases at the end-of-life stage. Some patients were directly admitted to a ward. More patients stayed in the AED of a tertiary/secondary hospital. From there, some patients were then transferred to a ward. The others remained in the AED until the end.

“If a patient does not die soon, we will try to transfer him/her to a ward for further treatment.” (Physician 2, AED)

Local medical insurance policies allow patients to stay in a tertiary hospital for 7 days, or in a secondary hospital for 21 days. This amount of time is insufficient for patients at this stage, so they are transferred from one location to another. The transfers can be arranged to take place within the same hospital or among hospitals.

“After a patient stays in a ward for three weeks, we can arrange a self-financed re-admission. This means that the patient continues to stay in the ward, but pay all expenses by themselves for one week. After they can use their medical insurance again, we discharge and re-admit the patient again. All of the administrative procedures are done in the hospital information system. The patient stays in our ward throughout this period.” (Nurse 3, pulmonary)

Regardless of which of the above arrangement is made, such transfers brought suffering to patients and their families.

“The patients had to keep moving. It was really painful for the patients and their families.” (Physician 1, hepatology)

In the end, apart from a small number of patients who died in community health care centers or at home, most patients died in tertiary/secondary hospitals, especially in AEDs. However AEDs were not a good place in which to die.

“Eight to nine hundred patients died in our AED each year, the majority was patients with a chronic disease. We usually have more than 100 patients stay in the AED hall. Most are dying. They lie on trolleys. There are no curtains between the trolleys. They spend their last days in a place like a refugee camp.” (Physician 12, AED)

Spending one’s last days in a hospital was common, but it was not a good way to die. The patients usually underwent worthless treatments. In a way, it was a waste of health care resources, because other patients who truly needed high-level treatment and highly technical facilities could not be admitted, due to the presence of these patients.

“For the patients, it was worthless.” (Physician 8, cardiology)

“For the tertiary hospitals, it was a waste of health care resources.” (Physician 1, hepatology)

#### Physiologically focused and excessive treatment

Treatment at the end-of-life stage may continue to focus on the primary disease. As the disease progresses, attention turns to the symptoms and complications of the disease, is described as “conservative” or “symptom-focused” treatment. Both kinds of treatments may continue until the end.

“We usually provide symptom-focused treatment, which is the main treatment in the last few days.” (Physician 12, AED)

Another change in treatment occurs after the patient is judged to be dying. Several treatments are used during the dying phase. In some wards, many drugs, including life-sustaining drugs, nutrition supplements, antibiotics, and even organ protective drugs, are used. Oxygen therapy and vital sign monitoring also continued to be delivered too. Only two participants mentioned the application of minimal treatment.

“I can only control the pain and prescribe oxygen therapy. Other treatments (i.e., life-sustaining drugs, antibiotics, resuscitation equipment, etc.) are unavailable here.” (Physician 10, community)

The physicians acknowledged the patients’ psychological needs, but could not do more.

“It is difficult to address this (psychological) area because of China’s health care system, or the doctors’ energy.” (Physician 8, cardiology)

#### Roles of patients and families in making treatment decisions

Few participants had heard about advanced directives. After judging a patient’s prognosis to be poor, the physician would initiate a conversation with the family to discuss future treatment. The essence of the conversation was to decide on whether to use invasive treatments and procedures. It was assumed that other treatments would continue to be delivered, but that they could be changed if the family proposed a change.

“Others, such as intravenous medications or nutritional support, would usually not be discussed, unless the family proposed it.” (Physician 5, nephrology)

In most cases, the family made the decisions without the patient’s participation. The family’s decision was influenced by many factors, such as the financial burden of the treatment, the perception of the patient’s clinical condition, age, societal norms, and so on.

“Some families continued treatment under the pressure of social judgment. They did not want to be blamed.” (Physician 14, ICU)

Only in a few special cases did the patients make their own decisions.

“We had (such a case) before. It was a young patient with cancer, who was able to communicate and had some medical knowledge. Only with such a patient could we have such a discussion (about the treatment).” (Physician 3, AED)

The physicians seldom discussed the patient’s treatment and prognosis with the patients themselves. Although patients sometimes spoke of giving up (i.e., terminating treatment), it was the decisions of their family that was finally followed. Under the circumstance of family-dominated decision making, a patient’s real wish could hardly be known.

“Definitely the doctors discuss it [treatment] with the families. We are not sure whether the families previously discuss it with the patients.” (Nurse 1, hepatology)

More than half of the families decided to terminate active treatment, while others chose resuscitation.

“Most gave up at last.” (Physician 15, cardiology)

Efforts to keep the patient alive occurred when the family had not yet decided what to do or when the family insisted on resuscitation. In a few cases, the patient was kept alive to give all members of the family time to arrive to “see the patient for one last time.” Such resuscitation only brought suffering to the patient, and consumed resources unnecessarily.

“We encountered such a situation. The family decided to give up. Then, the patient’s condition worsened at night. We used drugs and carried out cardiopulmonary resuscitation (CPR) to keep the patient alive in order to wait for all family members to come.” (Physician 10, community)

#### Instinctively enhanced nursing care

Although nurses were seldom involved in judging the end-of-life stage or dying phase, they instinctively provided more care to patients at the end-of-life stage.

“I can only try my best. If the environment in the room is not good, I try to keep the room clean and tidy, and more comfortable, and to keep extraneous people out of the room.” (Nurse 13, AED)

They made necessary adjustments on administration, caring for patients at a higher level of nursing care. For example, one nurse said, “We would pay extra attention to their basic nursing care” (Nurse 1, hepatology). Another nurse said, “We would have more intensive surveillance on them” (Nurse 3, pulmonary). Meanwhile, they tried to provide psychological support.

“I would hold the patient’s hand if the patient was conscious. I think it was a comfort for him/her.” (Nurse 8, pulmonary)

Only one community nurse claimed that some basic nursing care procedures were no longer followed when the patient was dying, in order to minimize disturbance to the patient. Nursing care after death was quite procedure-related. None of the participants mentioned the provision of bereavement support.

### Challenges, difficulties, and the future

#### Great pressure from families

The greatest challenge came from the families of the patients. Some families did not accept their loved one’s condition.

“The biggest challenge is the unwillingness of the family to accept the patient’s condition. Some people had quarreled with us. At that time, my work became very difficult.” (Physician 5, nephrology)

Sometimes, the physicians had to repeatedly explain a patient’s condition to his/her family. Some families contacted physicians with their personal interests in mind. The physicians had to deal carefully with these complicated families.

“Some families were involved in their own interests when discussing the treatment plan. For example, they wanted to inherit the patient’s apartment. These kinds of families were very difficult to communicate with.” (Physician 14, ICU)

Meanwhile, the nurses’ work became difficult because many families expressed mistrust, showed poor cooperation and a lack of understanding, and made unreasonable demands.

“The biggest challenge was the poor cooperation of the family. Last time, there was an elderly patient. We suggested that the family spend more time with the patient. But the family said, ‘You should take care of him since he is in the hospital’. Then they left.” (Nurse 10, cardiology)

Despite the participants' efforts, some families insisted on aggressive treatment. Worse, conflict and violence sometimes occurred after the patients died.

“There was a patient who died suddenly. The family thought the treatment was delayed because of us. They could not accept this and smashed things in the ward everywhere.” (Nurse 2, AED)

#### Practical difficulties in the delivery of care

Several difficulties in the delivery of care were also mentioned. A common difficulty was how to transfer patients out of a tertiary/secondary hospital after stabilization. Due to the lack of an effective referral system among health care institutions, physicians did not know where to place the patients.

“We don’t collaborate with nearby hospitals at lower levels. Therefore, it is difficult for us to transfer our patients out.” (Physician 2, AED)

The physicians also felt that it was a challenge to comfort patients who did not know their real condition.

“The family usually requests us to not tell the patient the truth. So it becomes very difficult to comfort the patient when his/her condition is deteriorating.” (Physician 1, hepatology)

For the nurses, the difficulties included an increased workload, great stress during the night shift, skill-related difficulties, and feeling powerless to solve family conflicts. A lack of effective interventions to manage symptoms or problems was another difficulty faced by the participants.

“The biggest difficulty was that some of their suffering could not be solved with my ability and skills” (Nurse 13, AED)

#### Urgent tasks in the future

The participants believed that some essential tasks need to be completed before local end-of-life care is developed in the future. The most important and repeatedly mentioned task, was educating the public on death, so that they would have the proper attitude towards it.

“I think it is the attitude towards death. The society holds an attitude of avoidance towards death. Many Chinese cancer patients did not know their diagnosis until they died. They did not have the chance to realize their wishes.” (Physician 14, ICU)

Meanwhile, the participants thought that government support was ultimately essential to develop local end-of-life care. Creating a better health care environment was another important but challenging task.

“There are too many conflicts between patients and health care providers. We become exceptionally self-protective and careful when caring for dying patients. It causes great stress. I hope the environment can improve.” (Nurse 16, nephrology)

#### End-of-life care model in the future

In terms of how patients at the end-of-life stage would best be cared for in the future, some participants stated that patients would be better cared for in non-palliative care settings, and that end-of-life care should be integrated into routine work.

“I think patients in tertiary hospitals still need this service. We may have one or two health care providers in each ward to deliver the care after training.” (Physician 1, hepatology)

Others thought that patients should be cared for in specialized end-of-life units in health care institutions at all levels. A few suggested that patients should be cared for at home. Regardless of the car model, there is a long road ahead in developing local end-of-life care service.

## Discussion

This study describes how health care providers in non-palliative care settings care for patients with life-threatening diseases at the end-of-life stage. The findings indicate a lack of general end-of-life care in non-palliative care settings in Shanghai. Although symptom management is practiced to certain extent, aggressive disease management, the overuse of medication in the last 48 h, and visits to emergency rooms are common, especially among patients with non-cancer chronic diseases [62–65]. The nursing care in this study has mainly physical and task-oriented, similar to the nursing care provided in other settings [66–68]. The participants in this study thought that the quality of death was unsatisfactory. However, since there have been few studies in China on the quality of death of patients, great effort is needed in the future to develop a localized scale to evaluate the quality of death and examine the level of satisfaction of the patients and their families.

Having conversations about a patient’s poor prognosis and final treatment plan is a difficult task for physicians, and such conversations are usually initiated at a very late stage in the study. This is similar to the practice of British physicians [69]. Having been trained in an atmosphere of active treatment for years, some doctors still think that patients should be actively treated until the end [69]. Others want to avoid deeper concerns [69], which they may not feel confident about discussing with patients and their families. Despite the similarities to other areas in the world, dying in a family-oriented society is more complicated. Traditional Chinese cultural values, i.e., filial piety and the power of the family, exert a deep influence on decision making at the end-of-life stage [70]. Chinese people feel “culturally obliged to provide every possible means to keep a patient alive” [71]. Accordingly, over-treatment at this stage is common. It is also common for family members to make medical decisions on behalf of the patient, even when the patient is conscious [72]. In the future, how to involve patients in end-of-life care, and to allow them to voice their wishes will be a great challenge in China.

The nurses interviewed in this study meet a number of patients at the end-of-life stage in their work. This suggests that end-of-life care is an area of practice for nurses working in general wards [73], but they are seldom involved in judging the end-of-life stage or the dying phase. Bloomer et al. [65] also found that nurses took a passive role in recognizing dying. The nursing care that was described in this study is still physical and task-oriented, although a certain amount of comfort care is given both physical and psychological. This is similar to the nursing care provided in other settings [67, 68]. High-quality end-of-life nursing care in non-palliative care settings is lacking.

Several challenges must be addressed before general end-of-life care can be developed in local settings. Fridh (2014) suggested that education about the end-of-life stage and a supportive environment are essential for providing end-of-life care [74]. These are exactly what are needed in Shanghai. A major obstacle to providing end-of-life care is that health care providers lack a clear idea of what end-of-life care means, as well as the relevant competencies. Relevant training is necessary to bring about change. With regard to patients and their families, the lack of a proper attitude towards death is a considerable obstacle in initiating end-of-life care in China. Chinese people usually hold a negative attitude towards death, even when the patient is dying [75]. Meanwhile, many Chinese people mistakenly believe that health care services are part of the general service industry [76]. They believe that health care providers must fix the patient’s problem as they requested, since they are paying for it. These factors also contribute to delayed discussions of final treatment plans and to the constant seeking of medical support in acute care hospitals. This places a great burden on hospitals and wastes valuable health care resources.

Much work needs to be done at the system level in Shanghai. A care pathway or special insurance policy should be considered, as this would allow patients to remain in one location without drifting among institutions. An end-of-life care network linking health care institutions at all levels should be considered as well. In recent years, new measures have been implemented to optimize health care services in China. For instance, medical treatment partnerships and community-based end-of-life care have been developed [77, 78]. These measures facilitate the management of chronic diseases and provide more beds for cancer patients at the end-of-life stage. However, end-of-life care should be considered in various settings. Additionally, the health care environment in China needs to be ameliorated. The tension between patients and the health care system over the years has had a significant impact on the work of health care providers, causing them great stress and even danger. It has made them particularly self-protective, which may ultimately compromise the quality of care provided to patients.

### Limitations

This study reveals the service gaps in local end-of-life care and identifies future directions for research. The participants were not invited from hospitals in each division, which would have provided a comprehensive representation of views. Using WeChat was a more efficient way to recruit participants, but, it may have compromised the representativeness of the study population. In addition, due to limited time and human resources, this study was conducted in one city. The experiences of health care providers in other areas and countries may differ.

## Conclusions

General end-of-life care is underdeveloped in China. As health care institutions in China, and around the world, are faced with increasing numbers of patients at the end-of-life stage with longer life expectancies, it is time to bring this topic to the forefront of health care service reform. An end-of-life service network that could benefit patients with life-threatening diseases, and that could be set up in institutions at all three levels, should be developed. The obstacles impeding the development of end-of-life care should be resolved before a comprehensive end-of-life care system is established. Training programs for health care providers and death education programs for the public should also be developed. In the future, more studies should be conducted to provide empirical evidence for designing localized end-of-life care programs, since patients with different diagnoses and from social backgrounds may have different preferences for end-of-life care.

## Additional file

Additional file 1: Illustration of the coding process. (DOCX 31 kb)

## Abbreviations

AED`: Acute and emergency department
COPD: Chronic obstructive pulmonary disease
CPR: Cardiopulmonary resuscitation
ICU: Intensive care unit
UK: The United Kingdom
US: The United States
