# Supplementary material for: The experience of caring for patients at the end-of-life stage in non-palliative care settings: a qualitative study
Source: BMC Palliat Care. 2018 Oct 17;17:116. doi: 10.1186/s12904-018-0372-7 (PMC6193297; doi:10.1186/s12904-018-0372-7)
Supplement: Supplementary file 1 — Illustration of the coding process. (DOCX 31 kb) [file 12904_2018_372_MOESM1_ESM.docx]

**Appendix** Illustration of the Coding Process

| **Theme** | **Sub-theme** | **Category** | **Sub-category** | **Code** |
| --- | --- | --- | --- | --- |
| Definition of the end-of-life stage | | Treatment change | | Changing to palliative treatment |
|  |  |  |  | Enhanced treatment |
|  |  | Increased frequency of admission | | Increased frequency of admission |
|  |  | Varied and unpredictable duration | | Heart failure: 3-6 months |
|  |  |  |  | Lung cancer: days - weeks |
|  |  |  |  | Cirrhosis: 6m-1year |
|  |  |  |  | Unpredictable |
|  |  | Criteria for specific diseases | | Heart failure: bed-bound all day |
|  |  |  |  | Renal failure: Creatinine > 400 |
| Health care in the end-of-life stage | Hospitalization: Drifting in the health care system | In AED | | Most |
|  |  |  |  | May be further transferred to an inpatient ward |
|  |  | Within-hospital transfer | | In-ward re-admission |
|  |  |  |  | To another ward for a temporary stay |
|  |  | Transfer among hospitals | | Family found a hospital |
|  |  |  |  | Via cooperative a relationship between physicians |
|  |  |  |  | Via official cooperation between hospitals |
|  |  |  |  | Experience of suffering |
|  |  | Locations of death | | In tertiary or secondary hospitals, especially in the AED |
|  |  |  |  | A few in community health care centers |
|  |  |  |  | Going home before death |
|  |  | The number of deceased patients | | 60-900 per year in the AED |
|  |  |  |  | Fewer in non-AED wards |
|  |  | Impacts | | A poor quality death |
|  |  |  |  | Waste of health care resources |
|  | Physiologically focused and excessive treatment | Continued treatment for primary disease | | Same as before |
|  |  |  |  | Might last until the end |
|  |  | Symptom-based treatment | | Life-sustaining support |
|  |  |  |  | Symptom control |
|  |  |  |  | Might last until the end |
|  |  | Treatment in last days | | Drugs used in last days |
|  |  |  |  | Oxygen therapy |
|  |  |  |  | VS monitoring |
|  |  |  |  | Not many treatments |
|  |  | Limited psychological support | | Important |
|  |  |  |  | No time to provide |
|  | Roles of patients and family in making treatment decision | Initiating conversation on the treatment plan | Prejudged that the prognosis was poor | Based on experience or intuition |
|  |  |  |  | After analyzing multiple factors |
|  |  |  | Topics of conversation | Treatment direction during hospitalization |
|  |  |  |  | Final resuscitation |
|  |  |  |  | Other treatment options were proposed by family |
|  |  | Family dominated the decision and the absence of the patient | Families decided in most cases | Respected the family's decision |
|  |  |  | Factors that affected family’s decision | Financial burden |
|  |  |  |  | Societal norms |
|  |  |  |  | Perception of patient's clinical condition |
|  |  |  |  | Patient's age |
|  |  |  | Special cases | By patient |
|  |  |  |  | By community staff and doctor |
|  |  |  | Absence of patient | Doctors won't talk with the patient |
|  |  |  |  | Spoke of giving up, but family's decision was followed |
|  |  |  |  | Communication between patient and family was unknown |
|  |  | Results of decision making | Documenting decision on medical chart | Decision on medical chart |
|  |  |  |  | Never signed by the patient |
|  |  |  |  | Signed each admission |
|  |  |  | More than half might give up | More than half might give up |
|  |  |  | A few decided on resuscitation | Family hadn't decided yet |
|  |  |  |  | Family insisted |
|  |  |  |  | Resuscitation after giving up |
|  |  |  |  | Impact |
|  | Instinctively enhanced nursing care | Not involved in judging the end-of-life stage or dying phase | | Not involved in judging the end-of-life stage or dying phase |
|  |  | Care principle | | More care in a naturally palliative way |
|  |  |  |  | Try one’s best in routine work |
|  |  | Adjustments on administration | | More visiting time in ICU |
|  |  |  |  | Arranged for a senior nurse to be present |
|  |  |  |  | Designating a farewell room |
|  |  | Cared at a higher level of nursing care | | Basic nursing was emphasized |
|  |  |  |  | More observations and monitoring |
|  |  |  |  | Exception: Reducing basic nursing care |
|  |  | More psychological support | | Integrated in routine work |
|  |  |  |  | Non-verbal support to patients and families |
|  |  |  |  | Verbal support |
|  |  | Nursing care after death | | Procedure-related |
| Challenges, difficulties, and the future | | Great pressure from family members | Physicians: Dealing with family about poor prognosis and death | Could not understand/accept the patient's condition |
|  |  |  |  | Their own interests were involved |
|  |  |  |  | Dealt carefully with family with ill thoughts |
|  |  |  | Nurses: Causing difficulties at work | Mistrust in health care providers |
|  |  |  |  | Poor compliance |
|  |  |  |  | Overly demanding |
|  |  |  |  | Unable to understand the nurses' work |
|  |  |  | Negative consequences | Conflict or violence occurred |
|  |  |  |  | Repeatedly explained the patient's condition |
|  |  | Practical difficulties in the delivery of care | Nowhere to transfer the patients out | Nowhere to transfer |
|  |  |  |  | Stress of turnover rate |
|  |  |  |  | Lack of an effective transfer system |
|  |  |  | Difficult to comfort the patients when the disease progressed | Difficult to comfort the patients when the disease progressed |
|  |  |  | Challenges faced by nurses | Great psychological stress of night shift |
|  |  |  |  | Increased workload |
|  |  |  |  | Procedure-related difficulties |
|  |  |  |  | Powerless to resolve family conflicts |
|  |  |  | Unable to manage symptoms or problems effectively | Unable to manage symptoms or problems effectively |
|  |  | Urgent tasks in the future | Death education to the public | Learn to accept death properly |
|  |  |  |  | Lack of proper attitude to death |
|  |  |  | Government support | Government support |
|  |  |  | Better health care environment | Better health care environment |
|  |  | End-of-life care model in the future | In non-palliative care settings | Location |
|  |  |  |  | Ward-based palliative care |
|  |  |  | In palliative care settings | Units in different health care institutes |
|  |  |  |  | Better care |
|  |  |  | Dying at home | Need good social support system |
